# Vanillic acid changed cucumber (*Cucumis sativus* L.) seedling rhizosphere total bacterial, *Pseudomonas* and *Bacillus* spp. communities

**DOI:** 10.1038/s41598-018-23406-2

**Published:** 2018-03-21

**Authors:** Xingang Zhou, Fengzhi Wu

**Affiliations:** 1 Department of Horticulture, Northeast Agricultural University, Harbin, China; 2 Key Laboratory of Biology and Genetic Improvement of Horticultural Crops (Northeast Region), Ministry of Agriculture, Harbin, China

## Abstract

Soil microorganisms are key drivers of plant productivity in terrestrial ecosystems, yet controls on their diversities and abundances are not fully elucidated. Phenolic acids, released through plant root exudation and residues decomposition, are usually referred as autotoxins of several crops, including cucumber. In this study, effects of vanillic acid (VA) on cucumber rhizosphere microbial communities were investigated by treating cucumber seedlings with VA every two days for five times. Amplicon sequencing, PCR-denaturing gradient gel electrophoresis and quantitative PCR were used to analyzed the 16S rRNA genes of total bacterial, *Pseudomonas* and *Bacillus* spp. communities. Results showed that VA at 0.05 μmol g^−1^ soil changed total bacterial community diversity and composition. In particular, VA inhibited the relative abundances of genera with plant-beneficial potentials, such as *Bacillus* and *Lysobacter* spp. Moreover, VA changed *Pseudomonas* and *Bacillus* spp. community compositions by altering the number and/or relative abundances of their OTUs; and decreased *Bacillus* spp. community abundance at 0.02 to 0.2 μmol g^−1^ soil and *Pseudomonas* spp. community abundance at 0.2 μmol g^−1^ soil. Overall, VA changed cucumber seedling rhizosphere total bacterial, *Pseudomonas* and *Bacillus* spp. communities, which maybe be associated with the adverse effects of VA on cucumber growth under soil conditions.

## Introduction

Allelopathy is the phytotoxicity of a compound or a group of compounds released from plant parts through leaching, root exudation, volatilization, or residue decomposition to susceptible plants^1^. Several plant species can inhibited the growth of its own kind through autotoxicity, a special kind of allelopathy^2^. In agricultural ecosystems, autotoxicity is implied as one of the main causing agents of soil sickness, the phenomenon of growth and yield reduction and disease increases when one crop repeatedly grown on the same land^2,3^. Autotoxicity can be overcame by several agricultural practices, such as crop rotation, selecting crop varieties resistant to autotoxins, and adopting a proper fallow period so that there is enough time for the decomposition of autotoxins^2^. Cucumber (*Cucumis sativus* L.), a popular vegetable with high economic importance that often continuously monocropped in the greenhouse, is vulnerable to soil sickness^4,5^. Previous studies demonstrated that phenolic acids (such as derivatives of cinnamic and benzoic acids) could exert detrimental effects on cucumber and were potential autotoxins of cucumber in both hydroponic solution and soil conditions^3,6,7^.

Soil microorganisms are responsible for the key processes associated with soil fertility and plant health, hence, greatly influence the functioning of terrestrial ecosystems^8^. Accumulating evidence suggests that changes in soil microbial communities may lead to alterations in the functions performed by the community, which can have a feedback effect on plant health and fitness^9^. Recent studies indicated that phenolic acids could act as specific substrates or signaling molecules for a large group of microbial species in the soil^10^. *In vitro* studies found that phenolic acids were able to influence the growth and physiological status of specific microorganisms, such as *Pseudomonas syringae*, *Fusarium oxysporum* and *Rhizoctonia solani*^3,11–13^. However, little information is available on how these acids can affect microbial communities in the soil^14,15^.

*Pseudomonas* and *Bacillus* spp. are ubiquitous in terrestrial ecosystems, and are frequently found in associations with plants, either as mutualists, saprophytes or pathogens. Particularly, some species of these two genera play major roles in nutrient mobilization, plant growth promotion and protection, and thus play an important role in agriculture^16,17^. For example, *P. putida*, *B. pumilus, B. subtilis* are able to promote cucumber growth, induce systemic resistance, and directly inhibit plant pathogens, such as *Fusarium oxysporum* f.sp. *cucumerinum*, a host-specific soil-borne pathogen of cucumber^18,19^. It has been shown that agricultural management regimes, including crop continuous monocropping, affected *Pseudomonas* and *Bacillus* spp. communities^16,20,21^. However, how phenolic acids affect soil *Pseudomonas* and *Bacillus* spp. communities are still not clear.

Previously, we observed that vanillic acid (4-hydroxy-3-methoxybenzoic acid, a dihydroxybenzoic acid derivative) accumulated in the soil after continuous monocropping of cucumber^22^. Vanillic acid also inhibited cucumber seedling growth and changed the whole bacterial community structure as evaluated by PCR-denaturing gradient gel electrophoresis (DGGE)^23^. However, detailed changes in rhizosphere microbial compositions are still unclear. In the present study, we further analyzed cucumber rhizosphere bacterial community with high-throughput sequencing technique, which can provide a higher resolution and a better understanding of environmental microbial communities than the PCR-based fingerprinting techniques^24^. Moreover, cucumber rhizosphere *Pseudomonas* and *Bacillus* spp. community structures and abundances were estimated by PCR-DGGE and quantitative PCR, respectively.

## Results

### Illumina Miseq sequencing data

In total, Illumina Miseq sequencing generated 21,2367 quality bacterial 16S rRNA gene sequences with an average read length of 396 bp. The Good’s coverage of each sample, which reflects the captured diversity, was larger than 98% (data not shown). Rarefaction curves of OTUs of all samples tended to approach the saturation plateau (Figure S1), which indicated that our sequencing data represented most of the total bacterial community composition.

### Bacterial Community Composition

**Figure 1:**
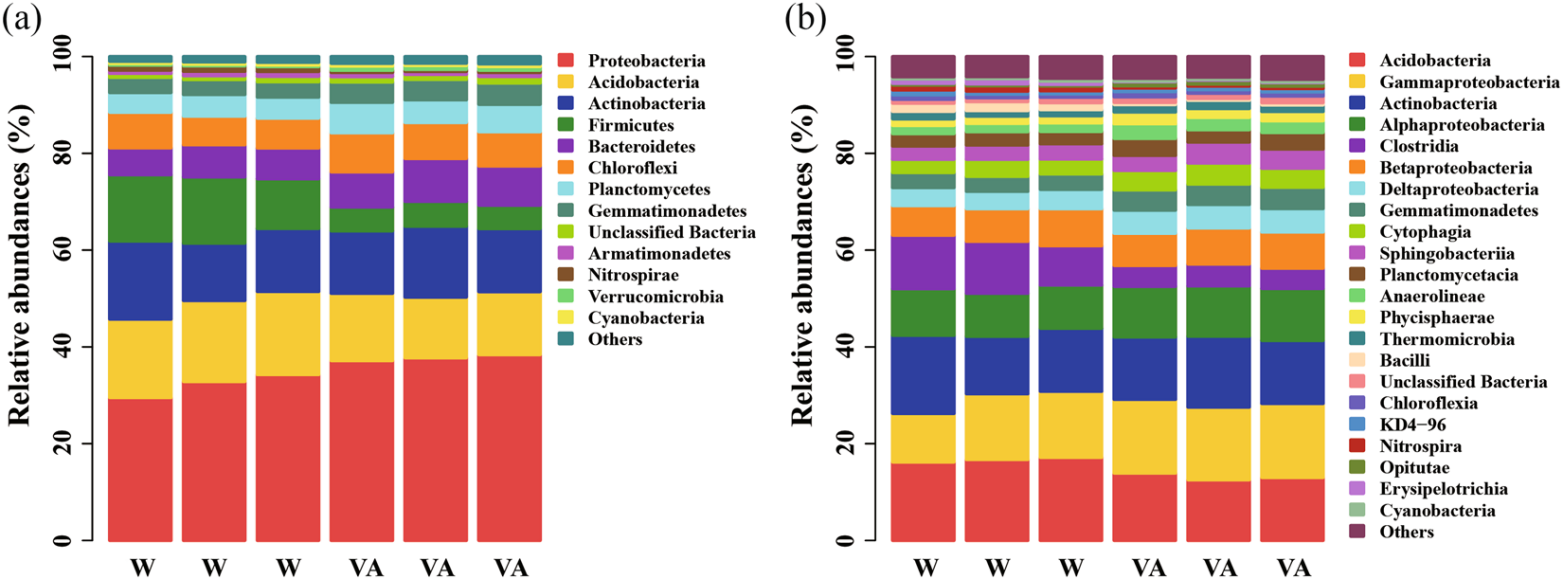
Relative abundances of main bacterial phyla (**a**) and classes (**b**) in cucumber rhizosphere soils treated with water (W) or vanillic acid at 0.05 μmol g^−1^ soil (VA). Bacterial phyla and classes with average relative abundances >0.5% in at least one treatment were shown. Data are represented as the means of three independent replicates.

**Figure 2:**
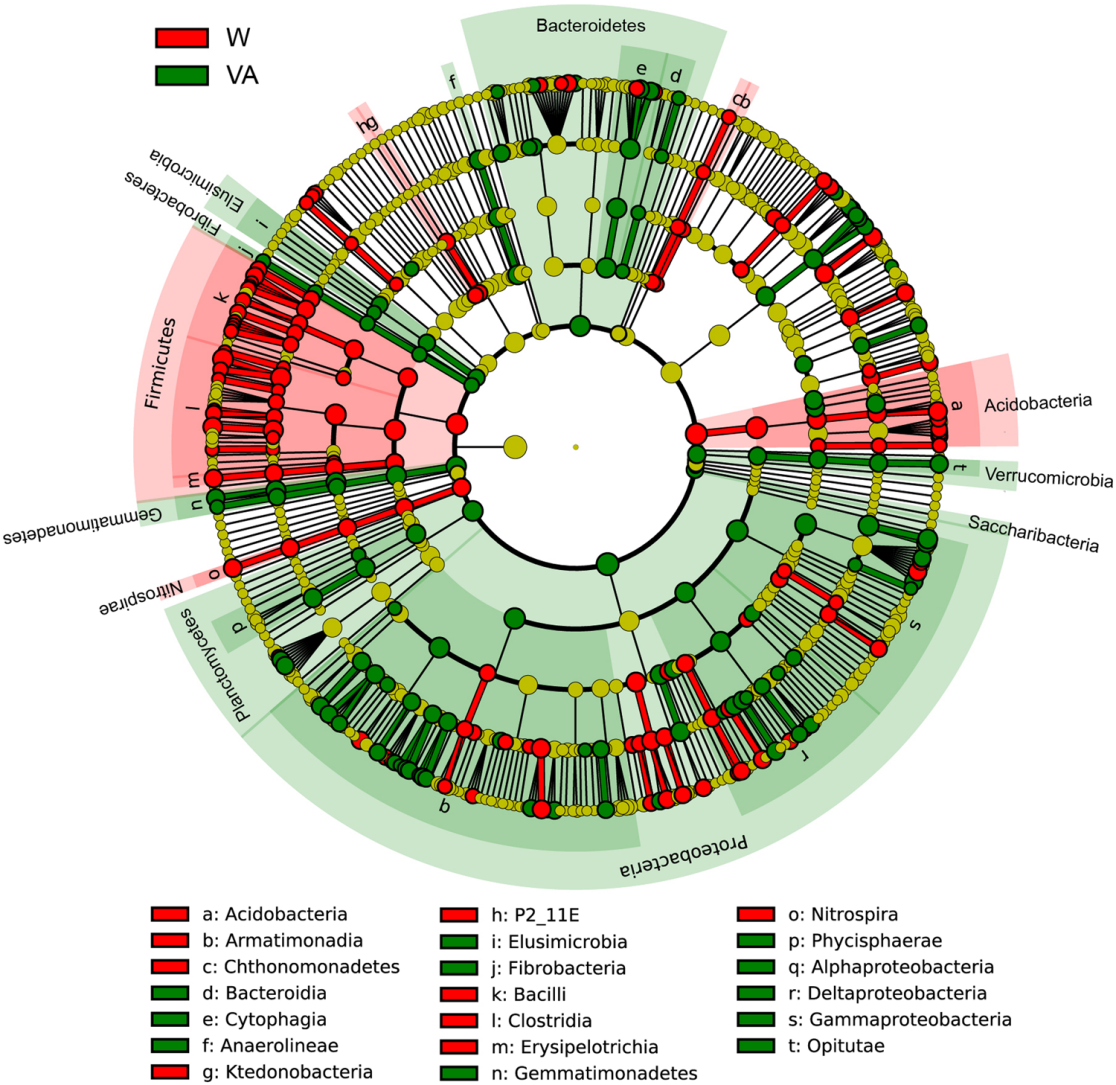
Cladograms, generated from LEfSe analysis, represent the polygenetic distribution of cucumber rhizosphere soil bacterial taxa. LEfSe analysis was based on the data of three independent replicates of each treatment. Bacterial taxa that are significantly enriched in each treatment with LDA scores larger than 2.0 are shown. Significantly discriminant taxon nodes are colored: red for cucumber rhizosphere soils treated with water (W), green for vanillic acid at 0.05 μmol g^−1^ soil (VA). Yellow circles represent non-significant differences in abundance between treatments for that particular taxon. Each circle’s diameter is proportional to the taxon’s abundance. Labels are shown of the phylum, class and order levels. The LDA scores of each significantly discriminant taxon from the phylum to genus levels are shown in Figure S2.

Across all samples, 32 bacterial phyla were detected and 1.06% of the bacterial sequences were unclassified at the phylum level. Proteobacteria, Acidobacteria, Actinobacteria, Firmicutes, Bacteroidetes and Chloroflexi were the dominant phyla (relative abundance > 5%), which accounted for more than 86% of the bacterial sequences (Fig. 1a). Compared with rhizosphere soils treated with water, rhizosphere soils treated with 0.05 μmol g^−1^ soil vanillic acid had higher relative abundances of Proteobacteria, Planctomycetes, Bacteroidetes, Gemmatimonadetes and Verrucomicrobia, but lower relative abundances of Acidobacteria, Firmicutes and Nitrospirae (P < 0.05) (Figs 1a, 2).

At the class level, more than 70 bacterial taxa were detected. The top three classes were Acidobacteria, Gammaproteobacteria and Actinobacteria, which accounted for about 42% of the bacterial sequences (Fig. 1b). Cucumber rhizosphere soils treated with 0.05 μmol g^−1^ soil vanillic acid had higher relative abundances of Alphaproteobacteria, Deltaproteobacteria, Gemmatimonadetes, Cytophagia, Anaerolineae and Opitutae, but lower relative abundances of Acidobacteria, Clostridia, Bacilli, Nitrospira and Erysipelotrichia (P < 0.05) (Figs 1b, 2).

**Figure 3:**
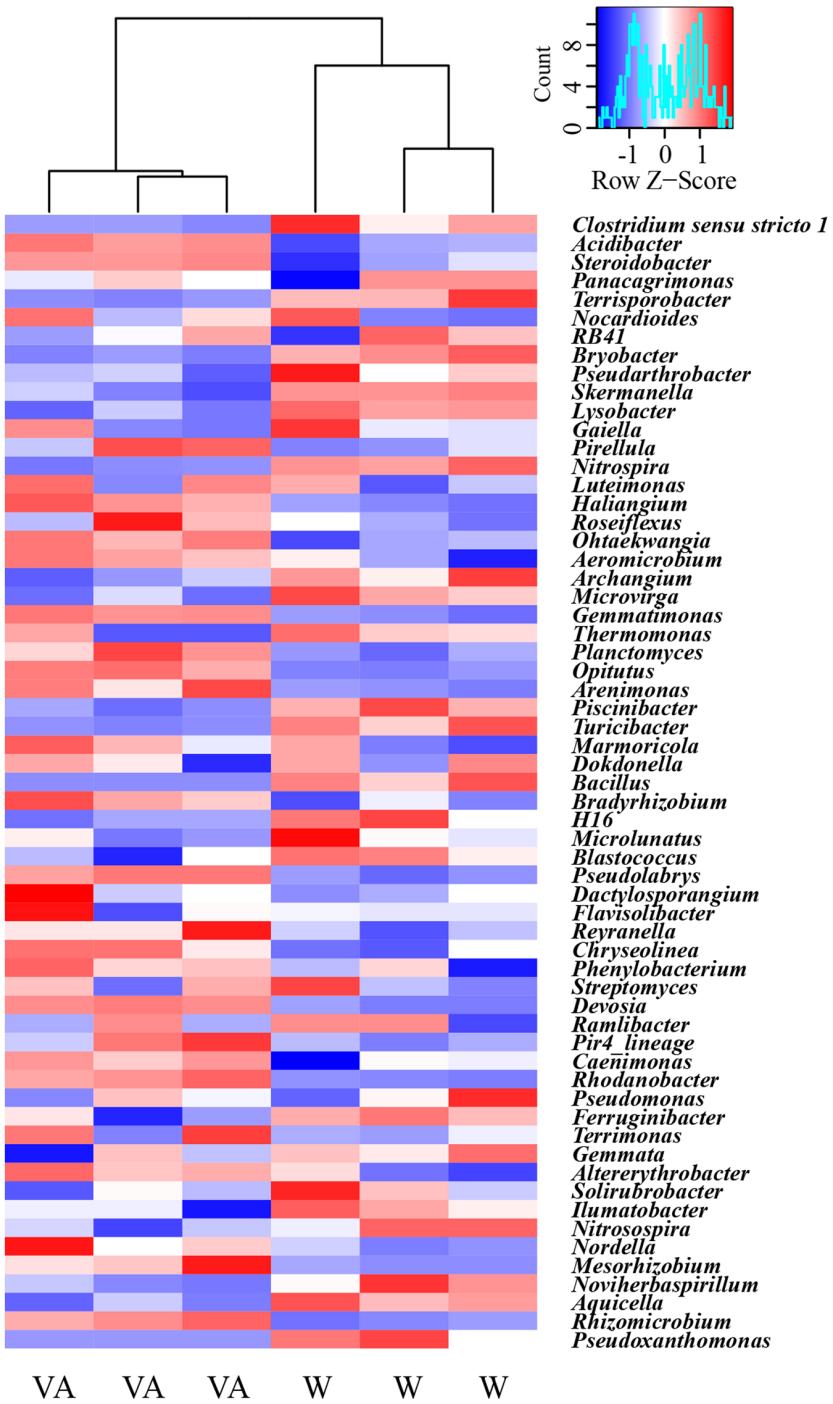
Heat map showing the relative abundances of dominant classified bacterial genera in cucumber rhizosphere soils treated with water (W) or vanillic acid at 0.05 μmol g^−1^ soil (VA). Classified bacterial genera with average relative abundances >0.3% were identified in each sample by colors deduced from the raw Z-scores. Hierarchical clustering of samples was performed using the average clustering method with the Euclidean distances.

At the genus level, more than 530 bacterial taxa were detected. *Gemmatimonas*, *RB41*, *Dokdonella*, *Bradyrhizobium*, *Nitrospira*, *Rhizomicrobium* and *Pseudolabrys* spp. were dominant classified genera (relative abundance > 1%) (Fig. 3). Cucumber rhizosphere soils treated with 0.05 μmol g^−1^ soil vanillic acid had higher relative abundances of *Acidibacter*, *Steroidobacter*, *Haliangium*, *Gemmatimonas*, *Opitutus*, *Ohtaekwangia*, *Pseudolabrys*, *Planctomyces*, *Arenimonas*, *Bradyrhizobium*, *Rhizomicrobium*, *Rhodanobacter*, *Devosia* and *Chryseolinea* spp., but lower relative abundances of *Terrisporobacter*, *Bryobacter*, *Turicibacter*, *Nitrospira*, *Bacillus*, *Skermanella*, *Piscinibacter*, *Lysobacter*, *Archangium*, *Microvirga*, *Noviherbaspirillum* and *Aquicella* spp. (P < 0.05).

A total of 2,060 OTUs were identified at 97% similarity across all samples. Most dominated OTUs (relative abundance > 0.5%) were mainly aligned to bacterial phylum Proteobacteria, Firmicutes, Acidobacteria, Bacteroidetes and Actinobacteria (Table 1). The relative abundances of seven OTUs, aligned to *Acidibacter*, *Steroidobacter*, Nitrosomonadaceae norank, Subgroup 7 norank, BIrii41 norank and *Bradyrhizobium* spp., were higher in rhizosphere soils treated with 0.05 μmol g^−1^ soil vanillic acid; while 11 OTUs, aligned to *Terrisporobacter*, *Clostridium sensu stricto 1*, Peptostreptococcaceae unclassified, *Archangium*, *Piscinibacter*, *Turicibacter*, SC-I-84 norank and *Bryobacter* spp. were higher in rhizosphere soils treated with water (P < 0.05).

**Table 1:** The most abundant bacterial OTUs in cucumber rhizosphere soils treated with water (W) or vanillic acid at 0.05 μmol g^−1^ soil (VA). OTUs were delineated at 97% sequence similarity. Only OTUs with average relative abundances >0.5% in at least one treatment were presented. Values were expressed as mean ± standard error (n = 3). OTU ID in bold indicates its relative abundance was significant different between treatments according to Welch’s *t* test (P < 0.05, Bonferroni corrected).

OTU ID	Phylum	Class	Genus	Relative abundances (%)	
				W	VA
**OTU1259**	Proteobacteria	Gammaproteobacteria	Acidibacter	1.75 ± 0.24	**3.72 ± 0.52**
OTU394	Firmicutes	Clostridia	Clostridium sensu stricto 1	3.14 ± 0.47	1.40 ± 0.52
OTU1239	Proteobacteria	Gammaproteobacteria	Panacagrimonas	1.99 ± 0.73	2.09 ± 0.11
**OTU1472**	Proteobacteria	Gammaproteobacteria	Steroidobacter	1.37 ± 0.13	**1.89 ± 0.12**
**OTU725**	Acidobacteria	Acidobacteria	Acidobacteria norank	**2.16 ± 0.12**	0.91 ± 0.36
**OTU429**	Firmicutes	Clostridia	Terrisporobacter	**2.18 ± 0.24**	0.85 ± 0.55
**OTU1435**	Firmicutes	Clostridia	Clostridium sensu stricto 1	**1.34 ± 0.13**	0.80 ± 0.09
OTU750	Acidobacteria	Acidobacteria	Acidobacteria unclassified	1.08 ± 0.06	1.05 ± 0.05
OTU914	Bacteroidetes	Cytophagia	Cytophagaceae norank	0.99 ± 0.02	0.97 ± 0.01
OTU1125	Actinobacteria	Actinobacteria	Pseudarthrobacter	1.15 ± 0.15	0.76 ± 0.06
**OTU906**	Firmicutes	Clostridia	Peptostreptococcaceae unclassified	**1.37 ± 0.20**	0.49 ± 0.37
OTU631	Acidobacteria	Acidobacteria	Acidobacteria unclassified	0.93 ± 0.03	0.79 ± 0.03
OTU1545	Firmicutes	Clostridia	Clostridium sensu stricto 1	1.05 ± 0.16	0.47 ± 0.17
OTU654	Acidobacteria	Acidobacteria	Acidobacteria norank	0.96 ± 0.06	0.54 ± 0.11
**OTU1754**	Proteobacteria	Betaproteobacteria	Nitrosomonadaceae norank	0.51 ± 0.04	**0.97 ± 0.10**
OTU1955	Proteobacteria	Gammaproteobacteria	Luteimonas	0.59 ± 0.05	0.74 ± 0.03
OTU301	Actinobacteria	Actinobacteria	Aeromicrobium	0.50 ± 0.09	0.74 ± 0.11
**OTU690**	Proteobacteria	Deltaproteobacteria	Archangium	**0.68 ± 0.03**	0.56 ± 0.05
**OTU1324**	Acidobacteria	Acidobacteria	Subgroup 7 norank	0.42 ± 0.08	**0.78 ± 0.11**
OTU1210	Proteobacteria	Gammaproteobacteria	Thermomonas	0.63 ± 0.02	0.55 ± 0.04
OTU665	Chloroflexi	Anaerolineae	Anaerolineaceae norank	0.56 ± 0.01	0.52 ± 0.01
OTU1321	Acidobacteria	Acidobacteria	Acidobacteria norank	0.49 ± 0.04	0.58 ± 0.08
**OTU148**	Proteobacteria	Betaproteobacteria	Piscinibacter	**0.68 ± 0.04**	0.38 ± 0.13
**OTU924**	Firmicutes	Erysipelotrichia	Turicibacter	**0.90 ± 0.11**	0.17 ± 0.28
**OTU1691**	Proteobacteria	Betaproteobacteria	SC-I-84 norank	**0.68 ± 0.03**	0.37 ± 0.14
OTU1282	Proteobacteria	Betaproteobacteria	Nitrosomonadaceae norank	0.45 ± 0.03	0.57 ± 0.05
OTU1127	Actinobacteria	Actinobacteria	Marmoricola	0.42 ± 0.09	0.59 ± 0.09
OTU1074	Bacteroidetes	Cytophagia	Cytophagaceae norank	0.45 ± 0.04	0.56 ± 0.05
**OTU660**	Proteobacteria	Deltaproteobacteria	BIrii41 norank	0.37 ± 0.04	**0.60 ± 0.05**
**OTU1983**	Acidobacteria	Acidobacteria	Acidobacteria norank	**0.57 ± 0.01**	0.39 ± 0.05
**OTU156**	Proteobacteria	Alphaproteobacteria	Bradyrhizobium	0.41 ± 0.03	**0.53 ± 0.03**
OTU1649	Actinobacteria	Actinobacteria	Microlunatus	0.53 ± 0.07	0.39 ± 0.04
**OTU1913**	Proteobacteria	Gammaproteobacteria	Steroidobacter	0.36 ± 0.03	**0.53 ± 0.06**
OTU1140	Actinobacteria	Actinobacteria	Blastococcus	0.51 ± 0.03	0.36 ± 0.08
**OTU704**	Acidobacteria	Acidobacteria	Acidobacteria norank	**0.53 ± 0.03**	0.32 ± 0.05
**OTU168**	Acidobacteria	Acidobacteria	Bryobacter	**0.58 ± 0.02**	0.24 ± 0.11

### Bacterial Community Diversity and Structure

**Figure 4:**
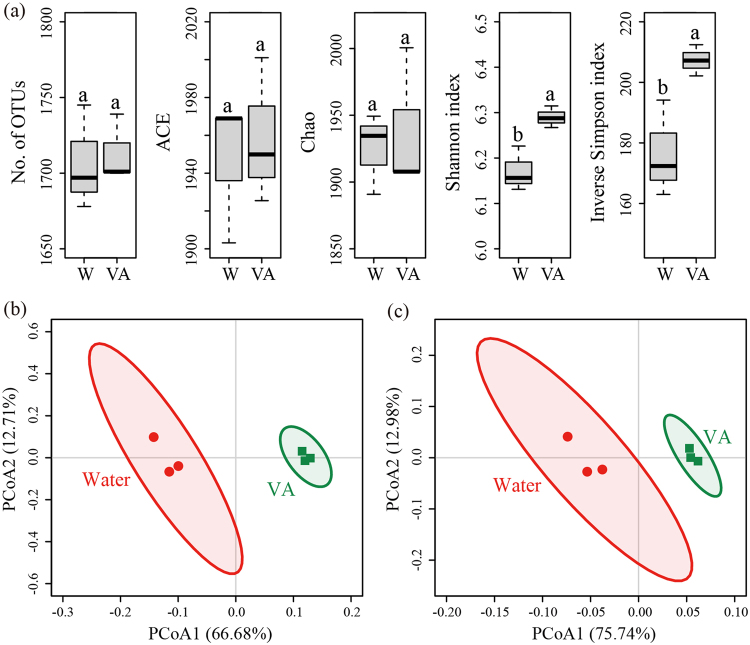
Alpha diversity and beta diversity indices of bacterial communities in cucumber rhizosphere soils treated with water (W) or vanillic acid at 0.05 μmol g^−1^ soil (VA). For alpha diversity, number of OTUs observed (No. of OTUs), ACE, Chao, Shannon and Inverse Simpson indices were calculated using random subsamples of 24,245 16S rRNA gene sequences per sample. OTUs were delineated at 97% sequence similarity. The box plot shows median (black line), first quartile–third quartile percentiles (box range) and 1.5× the interquartile range (whiskers). There were three independent replicates of each treatment. Different letters indicate significant difference based on Welch’s *t* test (P < 0.05). For beta diversity, differences in Bray-Curtis (**b**) and UniFrac distances (**c**) of bacterial communities at the OTU level were visualized by principal coordinates analyses. Ellipses indicate 95% confidence interval for replicates.

Rhizosphere soils treated with vanillic acid had higher Shannon and inverse Simpson indices than rhizosphere soils treated with water (P < 0.05) (Fig. 4a). However, number of OTUs, ACE and Chao indices were similar in rhizosphere soils treated with water and vanillic acid (Fig. 4a).

Principal coordinates analysis at the OTU level, based on both Bray-Curtis and UniFrac distance dissimilarities, revealed a clear separation between rhizosphere soils treated with water and vanillic acid (Fig. 4b,c).

### *Bacillus* and *Pseudomonas* spp. Community Compositions

Eight and five OTUs aligned to *Bacillus* and *Pseudomonas* spp., respectively, were detected in all treatments as determined by Illumina Miseq sequencing (Table S1). OTU275, OTU423 and OTU1458 were classified as *B. luciferensis*, *P. flexibilis* and *P. resinovorans*, respectively; while most of these OTUs could be aligned at the species level. For *Bacillus* spp., six OTUs were detected in rhizosphere soils treated with vanillic acid and eight OTUs in rhizosphere soils treated with water; the relative abundances of OTU275, OTU302 and OTU1222 were lower in rhizosphere soils treated with vanillic acid than in rhizosphere soils treated with water (P < 0.05). For *Pseudomonas* spp., the relative abundance of OTU1587 were higher in rhizosphere soils treated with vanillic acid than in rhizosphere soils treated with water (P < 0.05).

### *Bacillus* and *Pseudomonas* spp. Community Structures

**Figure 5:**
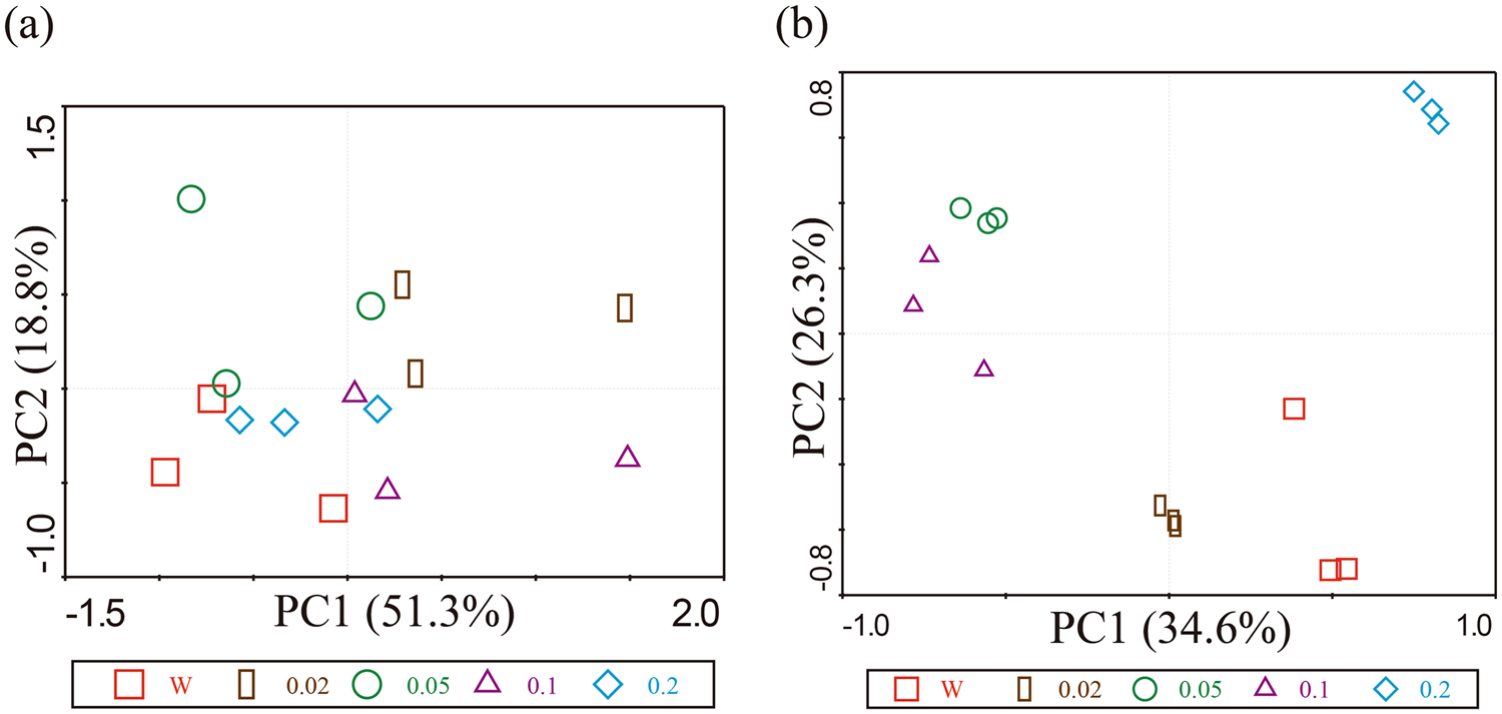
Principal component analysis of *Bacillus* (**a**) and *Pseudomonas* (**b**) spp. communities in cucumber rhizosphere soils based on PCR-DGGE analysis. W represents cucumber rhizosphere soils treated with water. 0.02, 0.05, 0.1 and 0.2 represent cucumber rhizosphere soils treated with vanillic acid at 0.02, 0.05, 0.1, 0.2 μmol g^−1^ soil concentrations, respectively.

For *Bacillus* spp. community, PCR-DGGE analyses showed that cucumber rhizosphere soils treated with water and different concentrations of vanillic acid (0.02, 0.05, 0.1, 0.2 μmol g^−1^ soil) had similar DGGE banding patterns (Figure S3a). Principal component analysis also could not clearly separate the five treatments from each other (Fig. 5a). Number of bands, Shannon-Wiener index and evenness index were not affected by exogenously applied vanillic acid (Figure S4).

For *Pseudomonas* spp. community, visual inspection of the DGGE profiles revealed different banding patterns among treatments (Figure S3b). Principal component analysis clearly distinguished the five treatments from each other (Fig. 5b). Compared with rhizosphere soils treated with water, rhizosphere soils treated with 0.2 μmol g^−1^ soil vanillic acid had lower number of bands, and rhizosphere soils treated with 0.02 μmol g^−1^ soil vanillic acid had higher Shannon-Wiener index and evenness index (Figure S4).

### *Bacillus* and *Pseudomonas* spp. Community Abundances

**Figure 6:**
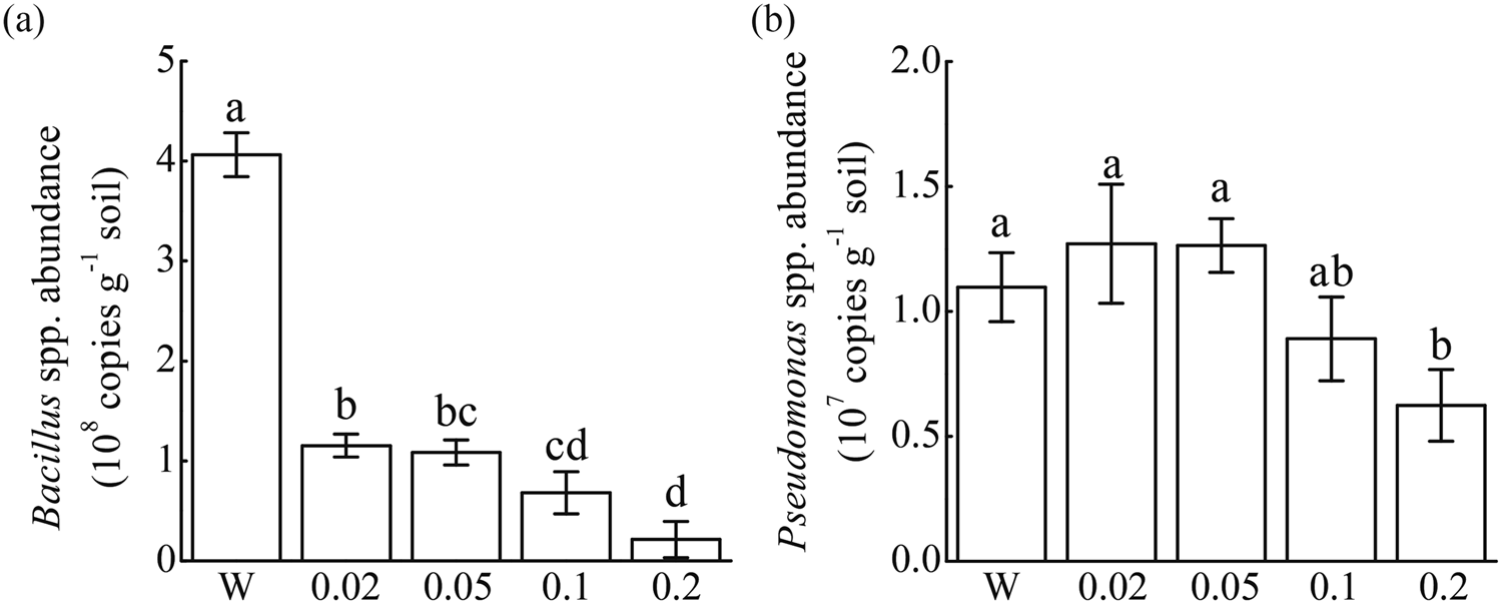
Abundances of *Bacillus* (**a**) and *Pseudomonas* (**b**) spp. communities in cucumber rhizosphere soils as determined by quantitative PCR. W represents cucumber rhizosphere soils treated with water. 0.02, 0.05, 0.1 and 0.2 represent cucumber rhizosphere soils treated with vanillic acid at 0.02, 0.05, 0.1, 0.2 μmol g^−1^ soil concentrations, respectively. Data are represented as the means of three independent replicates with standard error bars. Different letters indicate significant difference based on Tukey’s HSD test test (P < 0.05).

Quantitative PCR revealed that all concentrations of vanillic acid (0.02, 0.05, 0.1, 0.2 μmol g^−1^ soil) significantly decreased rhizosphere *Bacillus* spp. community abundance (P < 0.05) (Fig. 6a). The lowest *Bacillus* spp. community abundance was observed in rhizosphere soils treated with 0.2 μmol g^−1^ soil vanillic acid. Vanillic acid at 0.02, 0.05 and 0.1 μmol g^−1^ soil did not significantly affect cucumber rhizosphere *Pseudomonas* spp. community abundance (Fig. 6b). Vanillic acid at 0.2 μmol g^−1^ soil significantly decreased rhizosphere *Pseudomonas* spp. community abundance (P < 0.05). *Bacillus* and *Pseudomonas* spp. community abundances in rhizosphere soils treated with water was 19 and 1.76 times of these in rhizosphere soils treated with 0.2 μmol g^−1^ soil vanillic acid, respectively.

## Discussion

Plant-derived carbon metabolites, released through root exudation and residue composition, can be assimilated by soil microorganisms, the growths of which are usually limited by carbon resources^25^. It was observed that phenolic acids were rapidly decomposed by soil microorganisms after entering the soil^26,27^. Therefore, vanillic acid was applied into the soil periodically as described before^26^. Generally, the bioactivity of toxic compounds on plants and microorganisms was concentration dependent^28^. Previously, we found that the concentration of vanillic acid in cucumber rhizosphere was about 0.05 μmol g^−1^ soil in a continuous monocropping system^22^. In both natural and agricultural ecosystems, the concentration of soil phenolic acids was shown to range from 0.01 to 0.5 μmol g^−1^ soil^29–31^. Therefore, concentrations of vanillic acid used in this study (0.02 to 0.2 μmol g^−1^ soil) were within the realistic range of concentrations in the soil reported before.

Blum *et al*.^32^ found that phenolic acids inhibited cucumber seedling growth but stimulated the rhizosphere phenolic acid-utilizing bacteria. We also demonstrated that vanillic acid at concentration ≥0.05 μmol g^−1^ soil inhibited cucumber seedling growth and increased rhizosphere bacterial community abundance^23^. In this study, high-throughput amplicon sequencing of bacterial 16S rRNA gene was used to further illustrate the taxonomies of these responsive bacteria. Results showed that relative abundances of some bacteria such as *Rhodanobacter*, *Steroidobacter* and *Ohtaekwangia* spp., which have been reported to be involved in phenolic compounds degradation^33–35^, were enriched in cucumber rhizosphere soils treated with vanillic acid. Future *in vitro* studies should focus on validating the capabilities of these microorganisms to metabolize vanillic acid.

In continuous monocropping systems, plant usually showed reduced growth and vigor, and increased disease index^5,36^ For example, continuous monocropping of cucumber stimulated the abundance of *Fusarium oxysporum* f.sp. *cucumerinum*, the causing agent of cucumber Fusarium wilt disease^4^. Illumina MiSeq sequencing showed that, compared with rhizosphere soils treated with water, rhizosphere soils treated with vanillic acid (0.05 μmol g^−1^ soil) had higher relative abundances of *Bacillus*, *Archangium* and *Lysobacter* spp., which contained species to promote plant growth and inhibit plant pathogens^19,37,38^. Quantitative PCR also found that all concentrations of vanillic acid (0.02 to 0.2 μmol g^−1^ soil) decreased cucumber rhizosphere *Bacillus* spp. abundance. Some species in *Bacillus* spp. are able to inhibit plant soil-borne pathogens, including *Fusarium oxysporum* f.sp. *cucumerinum*, and promote cucumber growth^19^. Therefore, decreases in cucumber rhizosphere plant-beneficial microorganisms induced by vanillic acid may contribute to increased soil-borne diseases in the continuous monocropping system.

In this study, vanillic acid increased the relative abundances of *Arenimonas*^39^, *Gemmatimonas*^40^, *Haliangium*^41^, *Opitutus*^42^, *Pseudolabrys*^43^, *Steroidobacter*^44^ and *Rhodanobacter* spp.^45^, which contained taxa with denitrification capabilities, dissimilatory reduction of nitrate to nitrous oxide and N_2_. Meanwhile, vanillic acid decreased the relative abundance of *Nitrospira* spp.^46^, which had nitrification capabilities, oxidation of ammonia to nitrite. These results validated previous studies showing that phenolic acids were able to inhibit nitrification^47^ and some denitrifiers were able to use phenolic acids as carbon sources^48^. Nitrogen is one of the major limiting elements in agricultural ecosystems^49^. Our results indicated that there was possibility that phenolic acids could influence plant growth through regulating rhizosphere nitrogen transformations, such as inhibiting nitrification and promoting denitrification.

Illumina MiSeq sequencing and PCR-DGGE analyses showed that vanillic acid changed the structure and composition of cucumber rhizosphere *Pseudomonas* spp. community. Illumina MiSeq sequencing revealed that vanillic acid also changed the composition of cucumber rhizosphere *Bacillus* spp. community. Moreover, quantitative PCR analysis showed that vanillic acid decreased *Bacillus* spp. community abundance at 0.02 to 0.2 μmol g^−1^ soil, and decreased *Pseudomonas* spp. community abundance at 0.2 μmol g^−1^ soil. It has been shown that agricultural intensification could negatively influence soil *Pseudomonas* and *Bacillus* spp. communities^16,20,21,50^. For example, continuous monocropping of *Radix pseudostellariae* changed the community structure and decreased the abundance of soil *Pseudomonas* spp.^20^. Continuous monocropping of *Helianthus tuberosus* changed soil *Pseudomonas* and *Bacillus* spp. community structures^51^. It is well known that continuous monocropping can lead to accumulation of autotoxins, including phenolic acids, in the soil^6,22,48^. Thus, accumulation of phenolic autotoxins may be linked the effects of continuous monocropping on *Pseudomonas* and *Bacillus* spp. communities.

Plants can influence the diversity and composition of rhizosphere microbial communities through releasing root exudates^10^. Generally, rhizosphere microbial communities have higher abundances but lower diversities than those of the bulk soil^8^. It has been observed that artificially applied phenolic acids can alter the composition and diversity of soil microbial communities in absence of host plants^10,15^. Phenolic autotoxins can damage plant root and enhance ion leakage^52^ and these changes may also affect rhizosphere microbial communities. Therefore, besides its direct effects, vanillic acid may also indirectly changed cucumber rhizosphere microbial communities through its effects on physiological status of cucumber. These also suggest that rhizosphere and bulk microbial communities may respond differently to phenolic acids, which need to be further elucidated.

Phenolic acids are ubiquitous secondary metabolites in plants^2,6^. However, the composition of phenolic acids in root exudates or rhizosphere soils differed among crop species^2,7,22,31^. Liu *et al*.^15^ found that benzoic acid, a phenolic compound found in peanut (*Arachis hypogaea*) root exudates, increased the relative abundance of *Burkholderia* spp. in soil. However, vanillic acid did not affect the relative abundance of *Burkholderia* spp. in this study. These indicated that different phenolic acids may have different effects on soil microbial communities. Previous studies have also revealed the structure-function relationships of the phytotoxic and antimicrobial activities of phenolic acids^6,53^. For example, cinnamic acid derivatives had higher inhibitory effects than their corresponding benzoic acid derivatives on cucumber seedlings^54^. Phenolic acids with the hydroxyl group in the position *para* to the carboxyl side chain, such as benzoic acid, can be easily metabolized by the maize pathogen *Cochliobolus heterostrophus*; while phenolic acids lacking the *para*-hydroxy group or their *para-*methoxy substituted derivatives, such as ferulic acid, have higher antifungal activity^53^. These evidences may help to explain phenolic acids act as autotoxins of several crops though all plants releases phenolic acids.

## Conclusion

Overall, our results revealed that vanillic acid changed cucumber rhizosphere total bacterial, *Pseudomonas* and *Bacillus* spp. community compositions, and *Pseudomonas* and *Bacillus* spp. community abundances. In particular, Illumina MiSeq sequencing showed that vanillic acid at 0.05 μmol g^−1^ soil inhibited the relative abundances rhizosphere microorganisms with plant-beneficial potentials. Quantitative PCR analysis showed that vanillic acid decreased *Bacillus* spp. community abundance at 0.02 to 0.2 μmol g^−1^ soil, and decreased *Pseudomonas* spp. community at 0.2 μmol g^−1^ soil. It is clear that phenolic acids are toxic to plants^2,6,7,52^. Results obtained from the present study suggested that phenolic acids may also inhibit plant growth through changing rhizosphere microbial communities, which need to be further explored by evaluating the effects of phenolic acids on the functions of rhizosphere microbial communities and their relationships with plant performance.

## Materials and Methods

### Pot Experiment

The soil used in this experiment was collected from the upper soil layer (0–15 cm) of an open field in the experimental station of Northeast Agricultural University, Harbin, China (45°41′N, 126°37′E), which was covered with grasses and undisturbed for more than 15 years. The soil has a sandy loam texture, contained organic matter, 3.67%; available N, 89.02 mg kg^−1^; Olsen P, 63.36 mg kg^−1^; available K, 119.15 mg kg^−1^; EC (1:2.5, w/v), 0.33 mS cm^−1^; and pH (1:2.5, w/v), 7.78. Cucumber seedlings (cv. Jinlv 3) with two cotyledons were transplanted into pots contained 150 g soil and maintained in a greenhouse (32 °C day/22 °C night, relative humidity of 60–80%, 16 h light/8 h dark). There was one cucumber seedling per pot. No fertilizer was added to the soil.

Cucumber seedlings at the one-leaf stage were treated with different concentrations of vanillic acid (0.02, 0.05, 0.1, 0.2 μmol g^−1^ soil) every two days for five times as recommended before^26^. The solution pH was adjusted to 7.0 with 0.1 M NaOH solution, because the soil pH is widely accepted as a dominant factor that regulates soil microbial communities55. Cucumber seedlings treated with distilled water were served as the control. Soil water content was adjusted every two days with distilled water to maintain a constant weight of pots. There were five treatments (four concentrations of vanillic acid and one control) in total. Each treatment had five seedlings and was replicated three times.

### Rhizosphere Soil Sampling and DNA Extraction

Ten days after the first application of vanillic acid, cucumber rhizosphere soil samples were collected as described before^5^. Briefly, cucumber roots were gently removed from the pot, and soils loosely attached to cucumber roots were carefully removed by manual shaking. Then, soils tightly adhering to roots were removed from the root surface by a sterile brush and considered as rhizosphere soils. After sieving (2 mm), rhizosphere soil samples were stored at −70 °C. Samples from five plants in each replicate were combined to make a composite sample. There were three composite rhizosphere samples for each treatment.

Total soil DNA was extracted with the PowerSoil DNA Isolation Kit (MO BIO Laboratories, Carlsbad, USA) as per the manufacturer’s instructions. Each composite soil sample was extracted in triplicate and the extracted DNA solutions were pooled.

### High-throughput Amplicon Sequencing and Data Processing

Total rhizosphere soil bacterial community compositions were estimated with high-throughput sequencing on an Illumina MiSeq platform. Primer set of F338/R806 was used to amplify V3-V4 regions of the bacterial 16S rRNA gene as described before^4,56^. Both the forward and reverse primers also had a six-bp barcode unique to each soil sample. The PCR protocol was: 95 °C for 3 min; followed by 27 cycles of 95 °C for 30 s, 55 °C for 30 s, 72 °C for 45 s; and a final extension at 72 °C for 10 min. Each DNA sample was independently amplified in triplicate. Products of the triplicate PCR reactions were pooled and purified using an Agarose Gel DNA purification kit (TaKaRa, China). Then, purified amplicons were quantified by a TBS-380 micro fluorometer with Picogreen reagent (Invitrogen, USA), and mixed accordingly to achieve the equal concentration in the final mixture. The mixture was then paired-end sequenced (2 × 300) on an Illumina Miseq platform at Majorbio Bio-Pharm Technology Co., Ltd., Shanghai, China.

Raw sequence reads were de-multiplexed, quality-filtered, and processed using FLASH^57^ as described before^4^. Operational taxonomic units (OTUs) were delineated at 97% sequence similarity with UPARSE using an agglomerative clustering algorithm^58^. Then, a representative sequence of each OTU was taxonomically classified through BLAST in Ribosomal Database Project (RDP) database^59^. Chimeric sequences were identified and removed using USEARCH 6.1 in QIIME^60^. To avoid potential bias caused by sequencing depth, a random subsampling effort of 24,245 16S rRNA gene sequences per sample was performed for further analysis. The data set was deposited in the NCBI-Sequence Read Archive with the submission Accession Number SRP119631.

### PCR-DGGE Analysis

Semi-nested PCR protocols were used to amplify *Pseudomonas* and *Bacillus* spp. 16S rDNA fragments. Primer sets of PsF/PsR and GC-338F/518 R were used for the first and second round of PCR amplification of *Pseudomonas* spp.^16^, respectively; while BacF/BacR and GC-338F/518 R were used for *Bacillus* spp.^21^. The PCR protocol was: 95 °C for 5 min; followed by 28 cycles of 95 °C for 30 s, 65 °C for 30 s for PsF/PsR and BacF/BacR (56 °C for 45 s for GC-338F/518 R), 72 °C for 90 s; and a final extension at 72 °C for 10 min.

For DGGE analysis, 6% (w/v) acrylamide gel with 45–65% denaturant gradient was used for *Pseudomonas* and *Bacillus* communities^51^. The gel was run in a 1 × TAE (Tris-acetate-EDTA) buffer for 14 h under conditions of 60 °C and 80 V with a DCode universal mutation detection system (Bio-Rad Lab, LA, USA). After the electrophoresis, the gel was stained in 1:3300 (v/v) GelRed (Biotium, USA) nucleic acid staining solution for 20 min. DGGE profiles were photographed with an AlphaImager HP imaging system (Alpha Innotech Crop., CA, USA) under UV light.

### Quantitative PCR Assay

Abundances of *Pseudomonas* and *Bacillus* spp. communities were estimated by quantitative PCR assays with primer sets of PsF/PsR^16^ and BacF/BacR^21^, respectively, as described before^51,61^. The PCR protocol was: 95 °C for 5 min; followed by 30 cycles of 95 °C for 30 s, 65 °C for 30 s, 72 °C for 90 s; and a final extension at 72 °C for 10 min. Standard curves were made with a 10-fold dilution series (10^2^–10^8^) of plasmids containing 16S rRNA genes of *Pseudomonas* and *Bacillus* spp. from soil samples. Sterile water was used as a negative control to replace the template. All amplifications were done in triplicate. The specificity of the products was confirmed by melting curve analysis and agarose gel electrophoresis. The threshold cycle (*Ct*) values obtained for each sample were compared with the standard curve to determine the initial copy number of the target gene.

### Statistical Analysis

For Illumina Miseq sequencing data, the defined OTUs were used to calculate taxon accumulation curves with the ‘vegan’ package in ‘R’^62^. Alpha diversity indices, Chao, ACE, Shannon index and inverse Simpson index were calculated using QIIME^60^. For beta diversity analysis, weighted UniFrac distances and Bray-Curtis distances were calculated using QIIME^60^ and ‘vegan’ package in ‘R’^62^, respectively. Principal coordinates analysis was conducted to visualize the community similarity with the ‘vegan’ package in ‘R’^62^. Linear discriminant effect size (LEfSe) analysis was used to identify microbial taxa that were significantly associated with each treatment with an alpha value of 0.05 for the Kruskal-Wallis test and a threshold of 2.0 for logarithmic linear discriminant analysis (LDA) scores^63^. Differences in relative abundances of microbial taxa between treatments were analyzed using Welch’s *t* test with Bonferroni correction in ‘STAMP’^64^.

The DGGE profiles banding patterns were analyzed with Quantity One V4.5 (Bio-Rad Lab, LA, USA). Principal component analysis was used to compare the band patterns between samples with Canoco for Windows 4.5 software (Plant Research International, Wageningen, the Netherlands). The microbial community diversity indices, including number of bands, Shannon-Wiener index and evenness index, were calculated as described before^5^.

Data were analyzed by analysis of variance (ANOVA). For alpha diversity indices from Illumina Miseq sequencing, mean comparison between treatments was performed based on the Welch’s *t* test at the 0.05 probability level. For diversity indices from the PCR-DGGE analysis, mean comparison between treatments was performed based on the Tukey’s honestly significant difference (HSD) test at 0.05 probability level.

## Electronic supplementary material

Supplementary Information
