# Supplementary material for: Vanillic acid changed cucumber (Cucumis sativus L.) seedling rhizosphere total bacterial, Pseudomonas and Bacillus spp. communities
Source: Sci Rep. 2018 Mar 21;8:4929. doi: 10.1038/s41598-018-23406-2 (PMC5862977; doi:10.1038/s41598-018-23406-2)

**Vanillic acid changed cucumber (*Cucumis sativus* L.) seedling rhizosphere total bacterial, *Pseudomonas* and *Bacillus* spp. communities**

Xingang Zhou^1,2^, Fengzhi Wu^1,2^

^1^Department of Horticulture, Northeast Agricultural University, Harbin, China

^2^Key Laboratory of Biology and Genetic Improvement of Horticultural Crops (Northeast Region), Ministry of Agriculture, Harbin, China

Correspondence and requests for materials should be addressed to F.W. (fzwuneau@yahoo.com).

Figure captions:

**Figure S1.** Rarefaction curves of the number of operational taxonomic units (OTUs) for cucumber rhizosphere bacterial community in each sample. Random subsamples of 24,245 16S rRNA gene sequences per sample were used to generate the rarefaction curves. Each curve represents an independent sample of each treatment. OTUs were delineated at 97% sequence similarity.

**Figure S2.** The LDA scores of each identified biomarker from the phylum to genus levels in each treatment from the LEfSe analysis of cucumber rhizosphere soil bacterial community composition (P<0.05, LDA>2.0). LEfSe analysis was based on the data of three independent replicates of each treatment. For clarity, only detected taxa with relative abundance >0.3% in at least one treatment were shown. W and VA represent cucumber rhizosphere soils treated with water and vanillic acid at 0.05 μmol g^-1^ soil, respectively.

**Figure S3.** DGGE profiles of *Bacillus* (a) and *Pseudomonas* (b) spp. communities in cucumber rhizosphere soils. W represents cucumber rhizosphere soils treated with water. 0.02, 0.05, 0.1 and 0.2 represent cucumber rhizosphere soils treated with vanillic acid at 0.02, 0.05, 0.1, 0.2 μmol g^-1^ soil concentrations, respectively. In the profiles, each lane represents an independent sample of each treatment.

**Figure S4.** Number of visible bands (a), Shannon diversity index (b) and Evenness index (c) based on DGGE analysis of *Bacillus* and *Pseudomonas* spp. communities in cucumber rhizosphere soils. W represents cucumber rhizosphere soils treated with water. 0.02, 0.05, 0.1 and 0.2 represent cucumber rhizosphere soils treated with vanillic acid at 0.02, 0.05, 0.1, 0.2 μmol g^-1^ soil concentrations, respectively. Data are represented as the means of three independent replicates with standard error bars. Different letters indicate significant difference based on Tukey's HSD test test (P<0.05).

**Table S1.** Relative abundances of OTUs belong to *Bacillus* and *Pseudomonas* spp. as determined by Illumina MiSeq sequencing in cucumber rhizosphere soils treated with water (W) or vanillic acid at 0.05 μmol g^-1^ soil (VA). OTUs were delineated at 97% sequence similarity. Values were expressed as mean±standard error (n=3). OTU ID in bold indicates its relative abundance was significant different between treatments according to Welch’s *t* test (P<0.05, Bonferroni corrected).

|  | OTU ID | W | VA |
| --- | --- | --- | --- |
| *Bacillus* spp. | OTU70 | 0.14±0.03 | 0.03±0.00 |
|  | OTU74 | 0.05±0.02 | 0.02±0.01 |
|  | **OTU275** | **0.05±0.01** | 0.01±0.00 |
|  | **OTU302** | **0.37±0.02** | 0.10±0.00 |
|  | OTU641 | 0.12±0.02 | 0.04±0.01 |
|  | OTU637 | 0.02±0.00 | 0.01±0.00 |
|  | **OTU1222** | **0.01±0.00** | 0.00±0.00 |
|  | OTU1390 | 0.01±0.00 | 0.00±0.00 |
| *Pseudomonas* spp. | OTU423 | 0.05±0.02 | 0.01±0.00 |
|  | OTU1165 | 0.13±0.08 | 0.04±0.01 |
|  | OTU1429 | 0.12±0.02 | 0.19±0.02 |
|  | OTU1458 | 0.01±0.01 | 0.01±0.00 |
|  | **OTU1587** | 0.02±0.01 | **0.05±0.01** |

**Figure S1.**


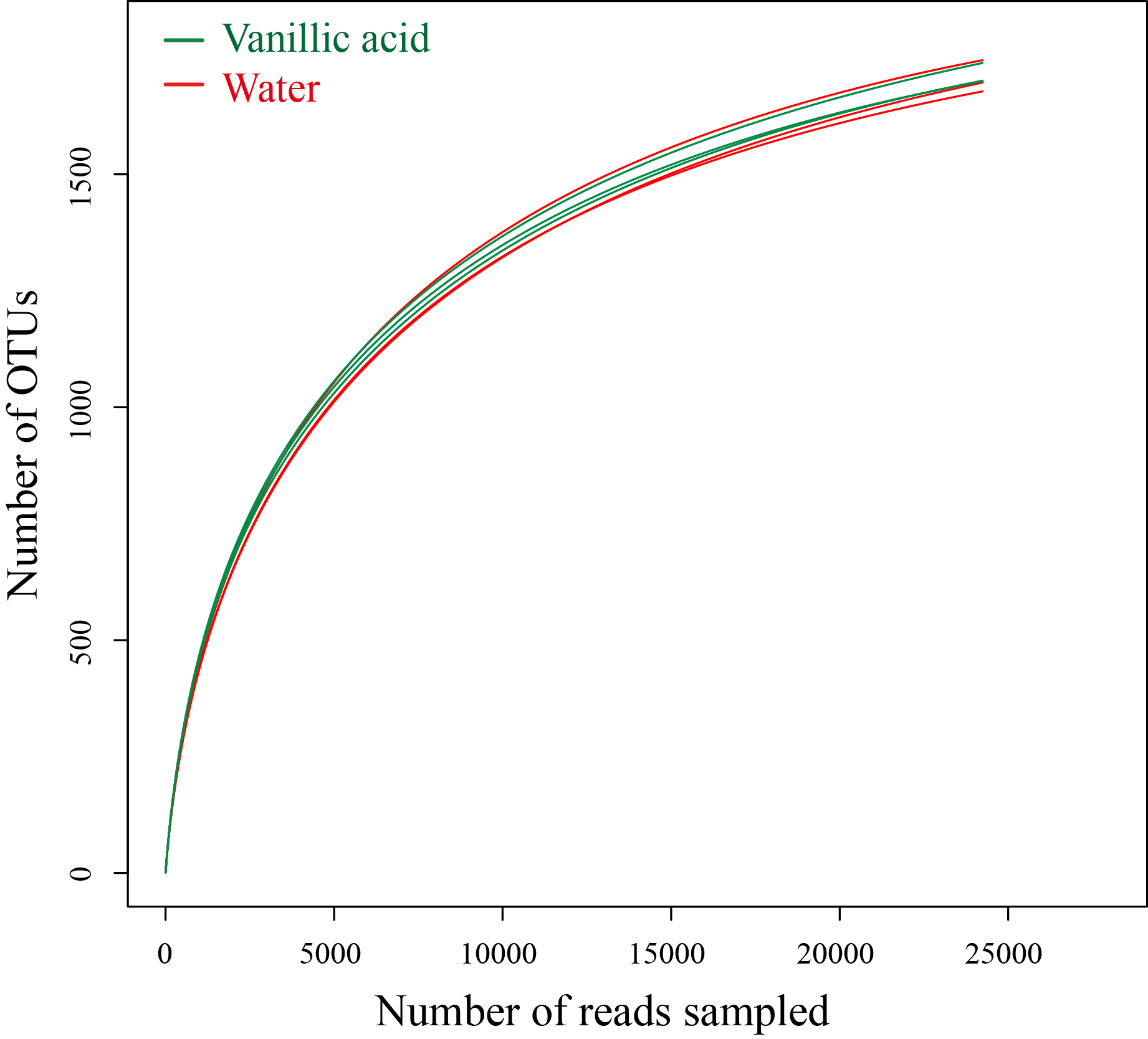


**Figure S2.**


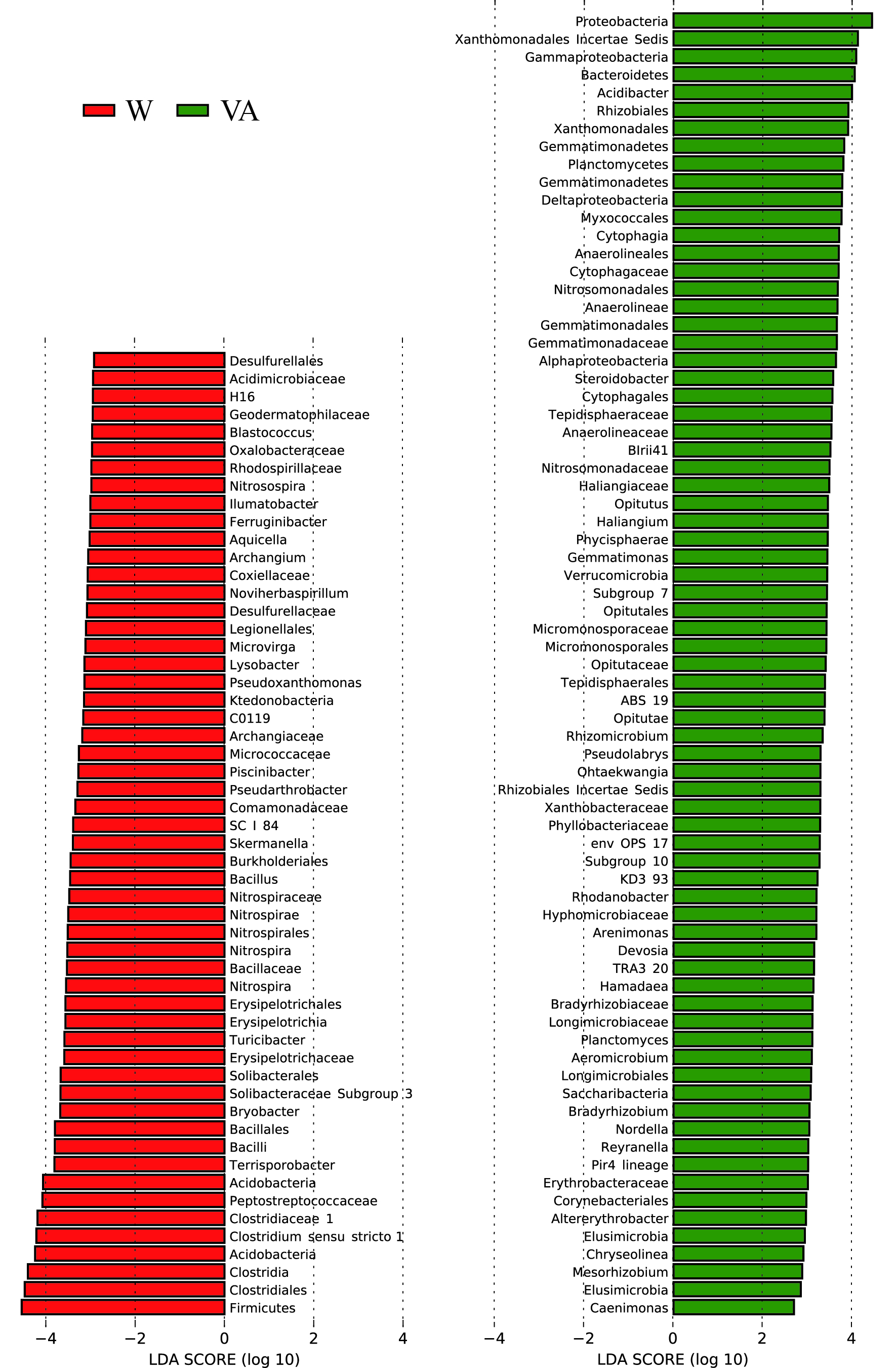


**Figure S3.**

**
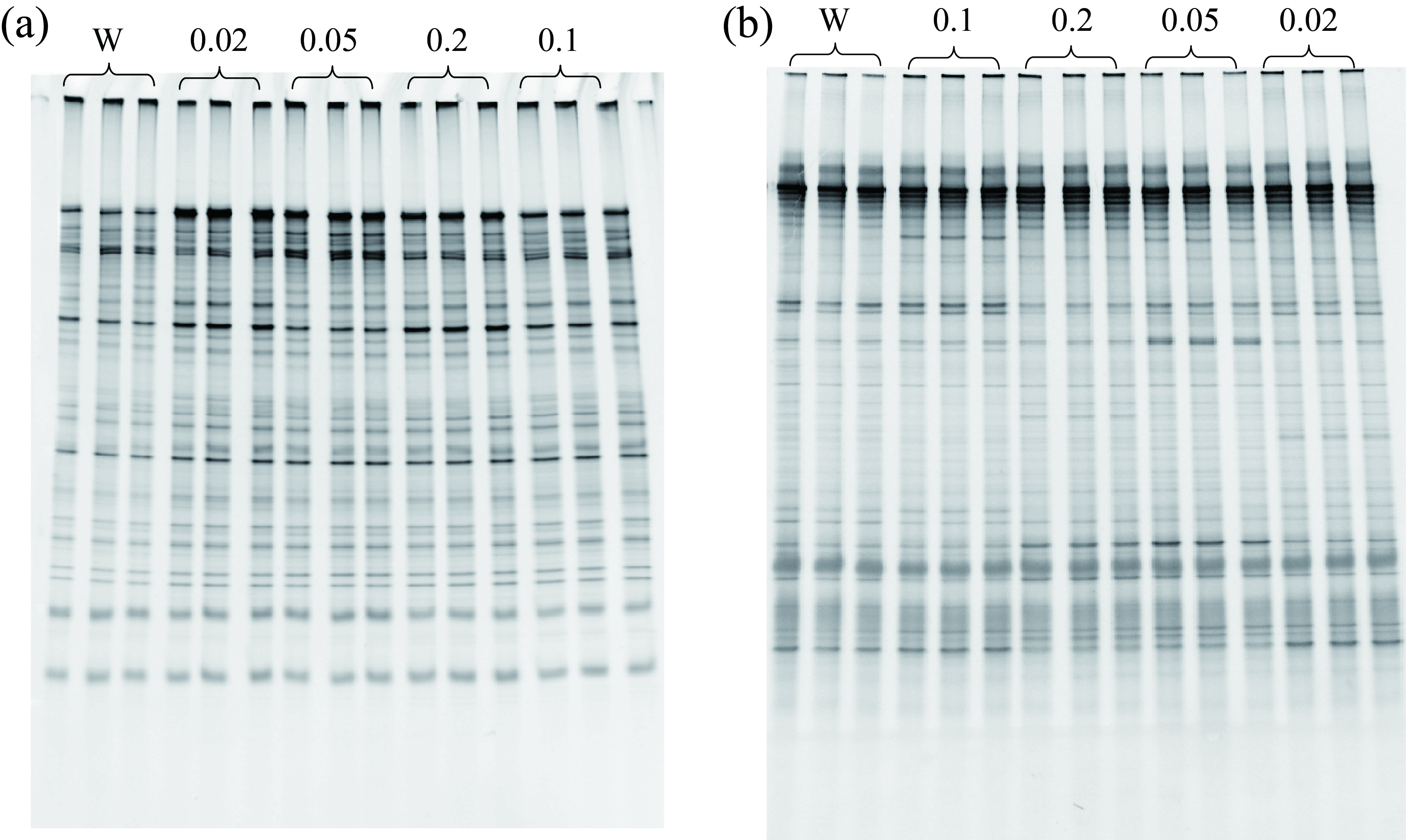
**

**Figure S4.**


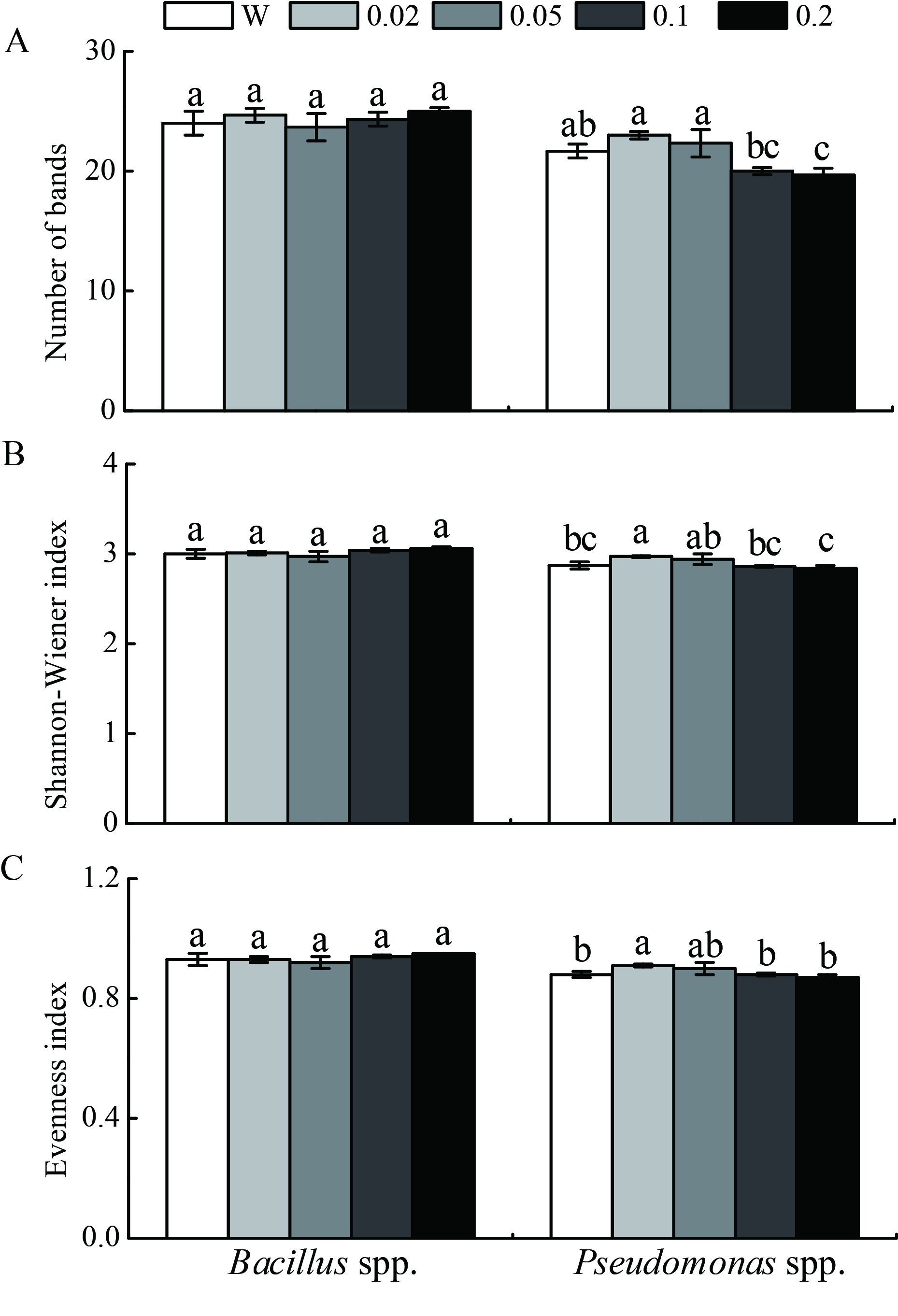

Supplement: Supplementary file 1 — Supplementary Information [file 41598_2018_23406_MOESM1_ESM.doc]
